# Clinical Impact of Multidrug-Resistant Bacterial Infections in Critically Ill Patients: A Retrospective Cohort Study

**DOI:** 10.3390/jcm15156110

**Published:** 2026-08-06

**Authors:** Mateusz Bartoszewicz, Ewa Płonowska, Marta Krysik, Jerzy Robert Ładny, Sławomir Lech Czaban

**Affiliations:** 1 Department of Anaesthesiology and Intensive Care, Medical University of Bialystok, Marii Skłodowskiej-Curie 24A St., 15-276 Białystok, Poland; ewa.plonowska@sd.umb.edu.pl (E.P.);; 2 Medical University of Bialystok, 15-276 Białystok, Poland; marta.krysik@icloud.com; 3 Department of Emergency Medicine, Medical University of Bialystok, Marii Skłodowskiej-Curie 24A St., 15-276 Białystok, Poland

**Keywords:** multidrug-resistant infection, intensive care unit, bacterial infection, mortality, mechanical ventilation, bloodstream infection, TISS-28

## Abstract

**Background/Objectives:** Antimicrobial-resistant bacterial infections are frequent in intensive care units (ICUs), but the independent effect of multidrug resistance relative to infection with susceptible organisms remains uncertain. We compared clinical characteristics, microbiology, treatment intensity, and in-hospital mortality among patients with no documented bacterial infection, antimicrobial-susceptible infection, or multidrug-resistant (MDR) infection. **Methods:** We conducted a single-center retrospective cohort study of the first eligible ICU hospitalization for each patient at the University Clinical Hospital in Bialystok, Poland, from 1 January 2017 to 1 June 2023. Infection groups were assigned using clinical documentation, microbiological results, and local laboratory resistance-phenotype coding; patients with multiple isolates were classified according to the most resistant clinically relevant isolate. Multivariable logistic regression evaluated associations with in-hospital mortality. **Results:** Among 3326 patients, 1413 (42.5%) had a documented bacterial infection: 481 (34.0% of infected patients) had susceptible infection and 932 (66.0%) had MDR infection. Crude mortality was 45.5%, 46.8%, and 48.4% in the no-infection, susceptible-infection, and MDR-infection groups, respectively (*p* = 0.354), whereas mean ICU length of stay was 10.0, 15.5, and 25.2 days (*p* < 0.001). In the complete-case whole-cohort model (*n* = 678), MDR infection (adjusted OR 2.70, 95% CI 1.84–3.96) and susceptible infection (adjusted OR 2.16, 95% CI 1.26–3.69) were associated with in-hospital death versus no infection. Within the infected cohort (*n* = 352), MDR status was not independently associated with mortality versus susceptible infection (adjusted OR 1.09, 95% CI 0.61–1.93). **Conclusions:** MDR infection identified patients with prolonged ICU exposure and greater organ-support requirements but did not independently increase mortality relative to susceptible infection. Interpretation is limited by the retrospective single-center design, complete-case analysis, and absence of time-resolved MDR acquisition data.

## 1. Introduction

Antimicrobial resistance (AMR) is a major public-health and development threat. In 2021, bacterial AMR was associated with an estimated 4.71 million deaths worldwide, including 1.14 million deaths directly attributable to resistance; the attributable burden is projected to reach approximately 1.91 million deaths annually by 2050 [1]. Critical care settings concentrate this risk. In the EPIC III point-prevalence study of 1150 ICUs, 54% of patients had suspected or proven infection, 70% received at least one antibiotic, and 22% had an ICU-acquired infection [2]. These findings illustrate the convergence of severe illness, invasive devices, extensive antimicrobial exposure, and healthcare-associated transmission in the ICU.

Multidrug-resistant (MDR) organisms reduce the probability of active empirical therapy, restrict definitive treatment options, and may necessitate broader-spectrum or combination regimens [3]. However, previous ICU studies have reported inconsistent estimates of the independent effect of MDR infection on mortality [4,5]. This heterogeneity may reflect differences in pathogen and infection-site case mix, reference-group selection, adequacy and timing of antimicrobial therapy, adjustment for severity of illness, and duration of ICU exposure. Analyses that treat resistance as a fixed exposure may also be affected by survivor or immortal-time bias, because patients must remain alive and hospitalized long enough to acquire many healthcare-associated resistant organisms [6,7,8].

The present analysis therefore used one integrated cohort with three mutually exclusive exposure groups: no documented bacterial infection, infection caused by antimicrobial-susceptible organisms, and MDR infection. Susceptible infection was defined as a clinically documented bacterial infection without a recorded acquired-resistance phenotype, whereas MDR infection was defined using the operational laboratory classification described in Section 2-Materials and Methods. Secondary analyses compared any bacterial infection with no infection, compared MDR with susceptible infection among infected patients, and evaluated factors associated with death in the infected cohort.

The objective was to determine whether MDR bacterial infection in critically ill patients was associated with distinct baseline characteristics, microbiological profiles, treatment intensity, and outcomes relative to susceptible infection and no documented bacterial infection. The primary outcome was in-hospital mortality. Secondary outcomes included ICU length of stay, mechanical ventilation, renal replacement therapy, vasoactive support, infection sites, pathogen distribution, and admission biomarkers.

## 2. Materials and Methods

### 2.1. Study Design, Setting, and Reporting Framework

This single-center retrospective cohort study was conducted in the adult ICU of the University Clinical Hospital in Bialystok, Poland. The source population comprised ICU admissions between 1 January 2017 and 1 June 2023. Data were obtained from routinely collected electronic hospital-, ICU-, and microbiology records. Reporting followed the Strengthening the Reporting of Observational Studies in Epidemiology (STROBE) framework [9]. The unit of analysis was the first eligible ICU hospitalization for each patient during the study period.

### 2.2. Participants and Cohort Flow

**Figure 1:**
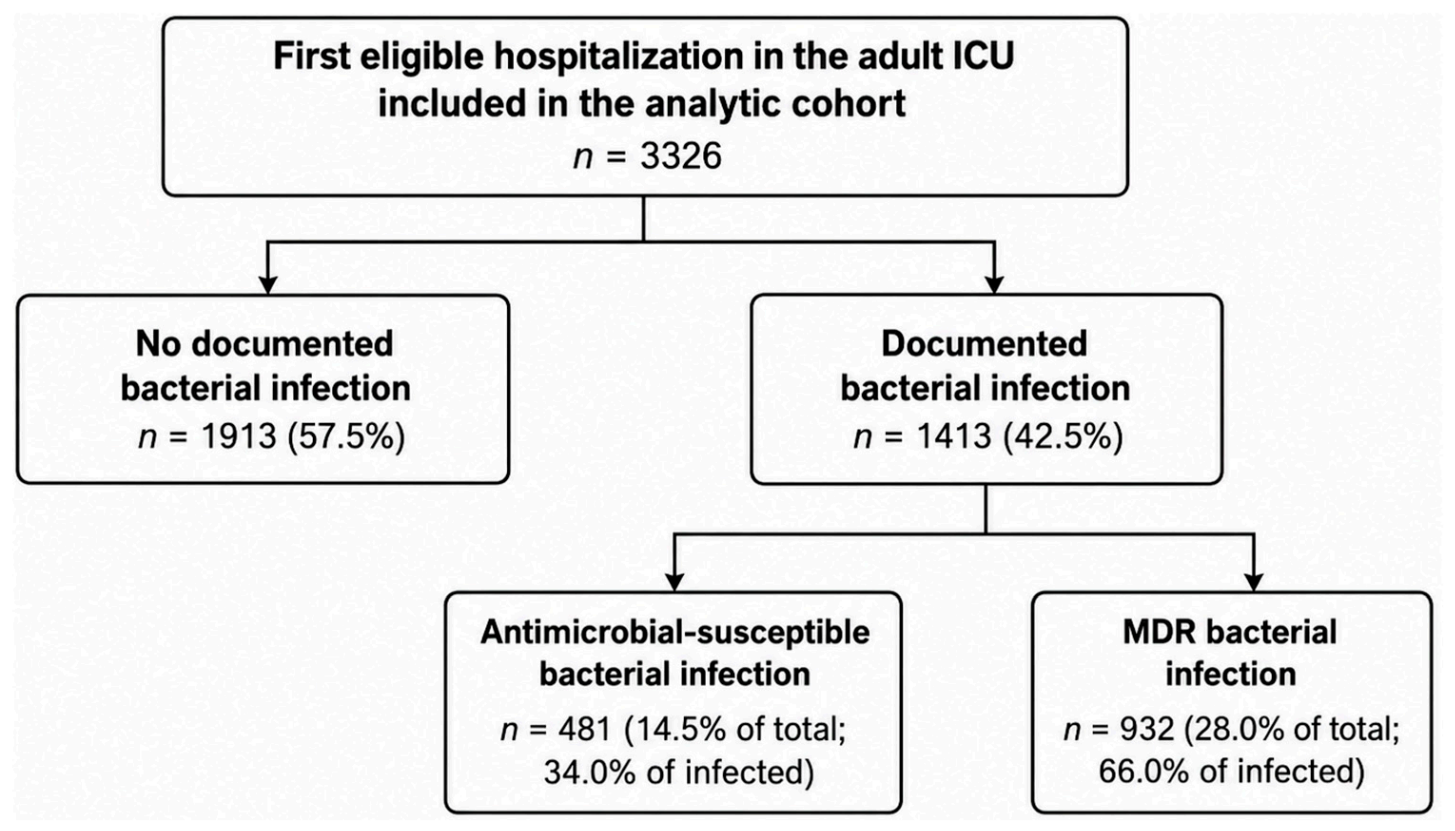
Patient flowchart and infection classification. Note. ICU, intensive care unit; MDR, multidrug-resistant.

Admissions to the adult ICU during the study period were eligible. For patients with more than one ICU hospitalization, only the first eligible hospitalization was retained to avoid correlated observations and repeated-measure bias. The final analytic cohort comprised 3326 index ICU hospitalizations. Patients were assigned to one of three mutually exclusive groups: no documented bacterial infection, antimicrobial-susceptible bacterial infection, or MDR bacterial infection. The cohort flow is presented in Figure 1.

### 2.3. Exposure Definition

The main exposure was the bacterial infection category assigned during the index ICU hospitalization. A documented bacterial infection required a clinically relevant microbiological result interpreted together with the treating team’s diagnosis; isolates documented as contaminants or colonization without infection were not classified as infections. Patients without a documented clinically relevant bacterial infection constituted the no-infection group. Infected patients with no recorded acquired-resistance phenotype constituted the susceptible-infection group. Bacterial pathogens were classified as sensitive, MDR based on the European Centre for Disease Prevention and Control definition [10].

The retrospective analytical extract did not retain complete agent-by-agent susceptibility panels for every isolate; therefore, resistance could not be reconstructed de novo across all antimicrobial categories. MDR status was operationalized from the hospital laboratory information system and required at least one clinically relevant isolate with a recorded acquired-resistance phenotype, including ESBL, MBL, or a specific carbapenemase marker, VRE, HLAR, MRSA, MLSB resistance, or MDR. Accordingly, the term MDR in this manuscript refers to this prespecified local MDRO/resistance-phenotype category interpreted in the context of the Magiorakos framework [10].

In polymicrobial infections and in patients with multiple clinically relevant isolates, group assignment was determined by the most resistant isolate recorded during the ICU stay. Pathogen and resistance-phenotype variables were otherwise non-mutually exclusive. Microorganism identification and antimicrobial susceptibility testing were performed by the hospital microbiology laboratory using routine diagnostic workflows. Minimum inhibitory concentration and susceptibility categories were interpreted according to the EUCAST breakpoints in force in the year of testing (versions 7.1–13.1 across 2017–2023) [11].

### 2.4. Outcomes

The primary outcome was in-hospital mortality. Secondary outcomes included ICU length of stay, duration and occurrence of mechanical ventilation, vasoactive drug use, renal replacement therapy, dialysis catheter insertion, artificial airway care, respiratory physiotherapy, enteral and parenteral nutrition, cardiopulmonary resuscitation, and other TISS-28 intervention indicators [12]. Microbiological secondary outcomes included pathogen distribution, resistance phenotypes, ventilator-associated pneumonia (VAP), bloodstream infection (BSI), and urinary tract infection (UTI).

### 2.5. Clinical Variables

Data were extracted retrospectively from electronic hospital and ICU records. Variables were grouped into demographic characteristics, comorbidities and clinical conditions, infection variables, treatments, admission laboratory parameters, gas-exchange variables, and TISS-28 intervention indicators. Comorbidities were identified from structured diagnosis fields and clinician documentation for the index hospitalization; the analytical extract did not contain a uniform ICD-10 code for every condition. Admission measurements were defined as values recorded at ICU admission or closest to the initial ICU assessment.

### 2.6. Infection-Site Variables

VAP, BSI, and UTI were analyzed as binary ICU infection-site variables. BSI was based on microbiological and clinical documentation, including clinically significant positive blood-culture results and physician-confirmed infection rather than isolated contamination. VAP was considered present when pneumonia was documented in a mechanically ventilated patient with compatible clinical, radiological, and microbiological findings. UTI was recorded when clinical documentation and microbiological evidence supported an ICU-acquired urinary infection diagnosis.

### 2.7. TISS-28 Intervention Variables

TISS-28 items were derived from daily ICU intervention documentation [12]. For each component, two variables were generated. First, a duration variable counted the number of ICU days on which the intervention was recorded. Second, a binary variable indicated whether the intervention was recorded at least once during the ICU stay. This approach separated prolonged exposure to routine ICU care from the occurrence of high-intensity rescue interventions.

### 2.8. Missing Data and Data Quality

Descriptive analyses used all available observations for each variable, and denominators therefore reflected variable-specific data availability. No imputation was performed. Multivariable regression analyses used complete cases for the variables included in each model. Missingness for the principal variables is reported in Supplementary Table S5, together with the model-specific complete-case sample sizes. Obesity was analyzed using the recorded binary BMI category; continuous BMI was not included in the analytical tables.

### 2.9. Statistical Analysis

Continuous variables are presented as mean (SD), and categorical variables as *n* (%). Unadjusted between-group comparisons used *t*-tests, analysis of variance, or non-parametric tests for continuous variables, as appropriate, and chi-square or Fisher exact tests for categorical variables. Pairwise standardized mean differences (SMDs) were calculated for selected baseline variables and are reported in Supplementary Table S6. Absolute mortality risk differences with 95% confidence intervals were calculated for the principal group comparisons. All tests were two-sided. Exact *p* values are reported to three decimal places; values below 0.001 are reported as *p* < 0.001.

Multivariable logistic regression evaluated adjusted associations with in-hospital mortality. Covariates were specified before model fitting on the basis of clinical relevance, plausible confounding, temporal availability at or near ICU admission, and data availability; they were not selected solely by univariable *p* values. The whole-cohort model used no documented bacterial infection as the reference category and included indicator variables for MDR and susceptible infection. The infected-cohort model compared MDR with susceptible infection. A separate prognostic model among infected patients incorporated selected infection-site and ICU-intervention variables. Variance inflation factors (VIFs) were examined in each model, with VIF < 5 interpreted as indicating no problematic multicollinearity. Results are reported as adjusted odds ratios (ORs) with 95% confidence intervals (CIs). Discrimination was summarized using accuracy, specificity, sensitivity, and area under the receiver-operating characteristic curve (AUC) at a probability cut-off of 0.5. Analyses were exploratory and represent adjusted associations rather than causal effects. Primary analyses were performed in Jamovi version 2.3 [13], with supplementary data-quality and effect-size calculations verified in Python 3.11.

### 2.10. Use of Generative Artificial Intelligence

ChatGPT, using the GPT-5.6 Pro model (OpenAI, San Francisco, CA, USA), was used as an AI-assisted writing tool during manuscript preparation to support English-language editing, improve clarity and consistency, and assist with manuscript organization and formatting. The tool was not used to collect data or independently conduct the statistical analyses; all analyses were performed with the software described in Section 2.9. All AI-assisted outputs were critically reviewed against the underlying study data and cited sources, edited by the authors, and approved by all authors, who take full responsibility for the final content.

## 3. Results

### 3.1. Cohort Flow and Infection Classification

The final analytic cohort comprised 3326 index ICU hospitalizations. Documented bacterial infection occurred in 1413 patients (42.5%), whereas 1913 (57.5%) had no documented bacterial infection during the ICU stay. Among infected patients, 481 (34.0%) had infection caused by susceptible organisms and 932 (66.0%) met the operational MDR definition. The MDR group represented 28.0% of the total cohort (Figure 1).

The primary comparison therefore included three groups: no documented bacterial infection, susceptible bacterial infection, and MDR bacterial infection. Baseline, intervention, microbiological and infection-site comparisons were unadjusted; adjusted mortality associations are reported separately.

### 3.2. Baseline Characteristics and Admission Profile

Age and sex distributions were similar across the three groups. Mean age was 63.7 years (SD 16.2) in the MDR group, 63.8 years (SD 16.3) in the susceptible-infection group, and 63.5 years (SD 16.8) in the no-infection group (*p* = 0.895). Female patients accounted for 36.2%, 39.3%, and 39.5% of the respective groups (*p* = 0.219).

Several baseline characteristics differed across infection categories. Obesity by BMI was recorded in 223 patients with MDR infection (23.9%) and 112 with susceptible infection (23.3%), compared with 320 patients without bacterial infection (16.7%; *p* < 0.001). COVID-19 was most frequent in the MDR group (170 patients, 18.2%), compared with the susceptible-infection group (31 patients, 6.4%) and the no-infection group (154 patients, 8.1%; *p* < 0.001). Antibiotic therapy during the preceding 3 months was uncommon but differed among groups (3.2%, 2.9%, and 1.6%, respectively; *p* = 0.011). Pressure ulcers were recorded in 8.2%, 4.6%, and 2.6% of patients, respectively (*p* < 0.001) (Table 1).

Admission CRP, white blood cell count, procalcitonin, and interleukin-6 did not differ significantly among the three groups (all *p* > 0.05). Creatinine was lower in both infected groups than in the no-infection group (1.5, 1.5, and 1.7 mg/dL; *p* = 0.014). Glucose and bicarbonate were highest in the MDR group (*p* = 0.008 and *p* < 0.001, respectively), whereas lactate was lower in the MDR and susceptible-infection groups than in the no-infection group (2.5, 2.9, and 3.6 mmol/L; *p* < 0.001) (Table 1). Pairwise SMDs for selected baseline variables are provided in Supplementary Table S6.

### 3.3. Primary Outcome and ICU Length of Stay

Crude in-hospital mortality did not differ significantly among the three infection categories. Death occurred in 451 patients with MDR infection (48.4%), 225 with susceptible infection (46.8%), and 871 without documented bacterial infection (45.5%; *p* = 0.354). The absolute mortality risk difference was 2.9 percentage points for MDR infection versus no infection (95% CI 1.1–6.8), 1.2 percentage points for susceptible infection versus no infection (95% CI 3.7–6.2), and 1.6 percentage points for MDR versus susceptible infection (95% CI 3.9–7.1) (Table 1).

ICU length of stay showed a marked gradient across the same three groups. Mean ICU stay was longest among patients with MDR bacterial infection (25.2 days, SD 21.6), intermediate among patients with susceptible bacterial infection (15.5 days, SD 16.2), and shortest among patients without bacterial infection (10.0 days, SD 24.8; *p* < 0.001). Thus, the MDR group had a mean ICU stay 9.7 days longer than the susceptible infection group and 15.2 days longer than the no-infection group (Table 1).

### 3.4. TISS-28 Intervention Duration and Treatment Intensity

TISS-28 duration variables showed consistently greater treatment exposure in patients with MDR infection. Mean duration of mechanical ventilation was 22.7 days (SD 19.6) in the MDR group, 13.3 days (SD 13.8) in the susceptible infection group, and 8.5 days (SD 11.0) in the no-infection group (*p* < 0.001). Similar gradients were observed for artificial airway care (26.4, 15.2, and 9.8 days; *p* < 0.001), respiratory physiotherapy (19.1, 12.1, and 4.9 days; *p* < 0.001), single vasoactive drug use (13.4, 8.1, and 4.1 days; *p* < 0.001), and central venous catheterization (27.3, 16.3, and 10.4 days; *p* < 0.001) (Table 2).

Renal and nutritional support also differed substantially. The mean duration of renal replacement therapy was 4.1 days in the MDR group, 2.2 days in the susceptible infection group, and 1.4 days in the no-infection group (*p* < 0.001). Parenteral nutrition lasted 11.1 days in the MDR group compared with 5.5 days in the susceptible infection group and 5.8 days in the no-infection group (*p* < 0.001). Enteral nutrition showed a similar pattern, with mean durations of 20.5, 11.6, and 6.9 days, respectively (*p* < 0.001). Procedures outside the ICU were also recorded for a longer duration in the MDR group (1.3 days) than in the susceptible infection group (1.0 days) or no-infection group (0.5 days; *p* < 0.001) (Table 2).

When TISS-28 items were analyzed as binary variables, most infected patients required invasive respiratory support. Mechanical ventilation was recorded at least once in 920 MDR patients (98.7%), 471 susceptible-infection patients (97.9%), and 1773 patients without bacterial infection (92.7%; *p* < 0.001). Artificial airway care occurred in 98.9%, 98.1%, and 93.6% of the three groups, respectively (*p* < 0.001). Single vasoactive drug use was more common in the MDR group (89.1%) than in the susceptible infection group (81.3%) and no-infection group (64.2%; *p* < 0.001). Multiple vasoactive drugs were recorded in 67.6%, 60.1%, and 62.4% of the groups, respectively (*p* = 0.006).

Other high-intensity ICU interventions were also more frequent in MDR infection. Cardiopulmonary resuscitation was recorded in 118 MDR patients (12.7%), 37 susceptible-infection patients (7.7%), and 222 patients without bacterial infection (11.6%; *p* = 0.017). Renal replacement therapy occurred in 29.2% of MDR patients, 22.0% of susceptible-infection patients, and 18.6% of patients without bacterial infection (*p* < 0.001). Dialysis catheter insertion was recorded in 24.9%, 15.6%, and 12.8% of these groups, respectively (*p* < 0.001). Parenteral nutrition and enteral nutrition were also more common in MDR infection than in the other groups (Table 2).

### 3.5. Concomitant ICU Infection Sites

Among the 1413 patients with bacterial infection, ventilator-associated pneumonia was the most frequent concomitant infection-site variable, occurring in 849 patients (60.1%). Bloodstream infection occurred in 374 infected patients (26.5%), and urinary tract infection occurred in 352 infected patients (24.9%).

The distribution of infection sites differed by MDR status. Ventilator-associated pneumonia was recorded in 545 patients with MDR infection (58.5%) and 304 with susceptible infection (63.2%; *p* = 0.097). Bloodstream infection was recorded in 320 (34.3%) and 54 (11.2%) patients, respectively (*p* < 0.001), and urinary tract infection in 270 (29.0%) and 82 (17.0%), respectively (*p* < 0.001). Thus, bloodstream and urinary tract infections were more frequent in the MDR group, whereas the frequency of ventilator-associated pneumonia did not differ significantly (Table 3).

### 3.6. Pathogen Distribution

The infected cohort had a broad microbiological spectrum comprising Gram-negative bacilli and Gram-positive cocci. The most frequently recorded organisms were *Klebsiella pneumoniae* (314 patients, 22.2%), *Staphylococcus aureus* (291, 20.6%), *Enterococcus faecalis* (261, 18.5%), *Pseudomonas aeruginosa* (211, 14.9%), *Escherichia coli* (205, 14.5%), *Klebsiella pneumoniae* subsp. *pneumoniae* (181, 12.8%), *Staphylococcus epidermidis* (158, 11.2%), and *Enterococcus faecium* (152, 10.8%) (Table 4).

Several pathogens differed in frequency between the MDR and susceptible-infection groups. *Klebsiella pneumoniae* was recorded in 30.4% versus 6.4% of patients, *Klebsiella pneumoniae* subsp. *pneumoniae* in 18.2% versus 2.3%, *Enterococcus faecalis* in 23.2% versus 9.4%, *Enterococcus faecium* in 14.7% versus 3.1%, *Pseudomonas aeruginosa* in 17.1% versus 10.8%, and *Staphylococcus epidermidis* in 15.6% versus 2.7% (all *p* ≤ 0.002).

*Escherichia coli*, *Hemophilus influenzae*, *Staphylococcus aureus*, *Hemophilus parahaemolyticus*, and *Streptococcus pneumoniae* were more frequent in the susceptible-infection group (all *p* ≤ 0.038). The main manuscript presents selected clinically relevant organisms; the complete pathogen distribution is provided in Supplementary Table S4.

### 3.7. Antimicrobial Resistance Phenotypes

Among the 932 patients with MDR bacterial infection, resistance phenotypes were not mutually exclusive. The most frequent were metallo-beta-lactamase (MBL; 281 patients, 30.15%), extended-spectrum beta-lactamase (ESBL; 274, 29.40%), MDR (a broad antimicrobial-resistance phenotype in the local coding system; 227, 24.36%), and high-level aminoglycoside resistance (HLAR; 197, 21.14%). Constitutive macrolide-lincosamide-streptogramin B resistance (MLSBK) was recorded in 149 patients (15.99%) (Table 5).

Carbapenemase-related markers included New Delhi metallo-beta-lactamase (NDM; 77 patients, 8.26%), Verona integron-encoded metallo-beta-lactamase (VIM; 11, 1.18%), oxacillinase-48-like carbapenemase (OXA-48; 8, 0.86%), and *Klebsiella pneumoniae* carbapenemase (KPC; 7, 0.75%). Vancomycin-resistant *Enterococcus* was recorded in 67 patients (7.19%), and methicillin-resistant *Staphylococcus aureus* in 41 (4.40%). Because phenotype categories were non-mutually exclusive, percentages should not be summed.

### 3.8. Secondary Simplified Analysis: Bacterial Infection Versus No Bacterial Infection

In the secondary comparison, crude in-hospital mortality was 47.8% among patients with bacterial infection and 45.5% among patients without documented bacterial infection (*p* = 0.199), corresponding to an absolute risk difference of 2.3% points (95% CI −1.1 to 5.7). Mean ICU length of stay was 21.9 versus 10.0 days, respectively (*p* < 0.001).

Patients with bacterial infection had longer recorded durations of several TISS-28 interventions than patients without documented infection: mechanical ventilation (19.5 versus 8.5 days), artificial airway care (22.6 versus 9.8 days), single vasoactive drug use (11.6 versus 4.1 days), central venous catheterization (23.5 versus 10.4 days), renal replacement therapy (3.5 versus 1.4 days), parenteral nutrition (9.2 versus 5.8 days), and enteral nutrition (17.5 versus 6.9 days) (all *p* < 0.001). Binary intervention variables showed the same direction for major respiratory, renal, and nutritional support measures (Supplementary Table S1).

Infection-site variables also distinguished infected from non-infected patients, as expected by definition and clinical documentation. Ventilator-associated pneumonia was recorded in 60.1% of patients with bacterial infection and 3.5% of patients without bacterial infection (*p* < 0.001). Urinary tract infection occurred in 24.9% and 2.3% of the two groups, respectively (*p* < 0.001), and bloodstream infection occurred in 26.5% of infected patients and none of the patients classified as having no bacterial infection (*p* < 0.001) (Supplementary Table S3).

### 3.9. Subgroup Analysis Among Infected Patients: MDR Versus Sensitive Infection

Among infected patients, crude in-hospital mortality was similar in the MDR and susceptible-infection groups (48.4% versus 46.8%; *p* = 0.604), with an absolute risk difference of 1.6 percentage points (95% CI −3.9 to 7.1). Time to death or end of observation was longer in the MDR group (23.3 days, SD 23.7) than in the susceptible-infection group (13.5 days, SD 17.8; *p* < 0.001).

Within the infected cohort, MDR infection was associated with longer duration of organ support and ICU care. Compared with susceptible infection, MDR infection had longer mechanical ventilation (22.7 versus 13.3 days; *p* < 0.001), artificial airway care (26.4 versus 15.2 days; *p* < 0.001), single vasoactive drug use (13.4 versus 8.1 days; *p* < 0.001), multiple vasoactive drug use (6.1 versus 3.7 days; *p* < 0.001), central venous catheterization (27.3 versus 16.3 days; *p* < 0.001), renal replacement therapy (4.1 versus 2.2 days; *p* < 0.001), parenteral nutrition (11.1 versus 5.5 days; *p* < 0.001), and enteral nutrition (20.5 versus 11.6 days; *p* < 0.001) (Supplementary Table S2).

The binary intervention analysis reinforced this pattern. Single vasoactive drug use occurred in 89.1% of MDR patients and 81.3% of susceptible-infection patients (*p* < 0.001). Multiple vasoactive drugs were recorded in 67.6% and 60.1% of the two groups, respectively (*p* = 0.006). Cardiopulmonary resuscitation was more frequent in MDR infection than in susceptible infection (12.7% versus 7.7%; *p* = 0.006), as were renal replacement therapy (29.2% versus 22.0%; *p* = 0.005), dialysis catheter insertion (24.9% versus 15.6%; *p* < 0.001), parenteral nutrition (66.4% versus 48.2%; *p* < 0.001), and enteral nutrition (87.8% versus 81.7%; *p* = 0.003).

Selected clinical variables also differed within the infected cohort. COVID-19 was more common in MDR infection than in susceptible infection (18.2% versus 6.4%; *p* < 0.001). Antifungal drug exposure (30.8% versus 21.6%; *p* < 0.001), steroid therapy (34.4% versus 28.3%; *p* = 0.022), and pressure ulcers (8.2% versus 4.6%; *p* = 0.016) were more frequent in the MDR group. In contrast, antibacterial drug exposure was recorded more frequently in the susceptible infection group (64.4%) than in the MDR group (58.6%; *p* = 0.037). Admission CRP, white blood cell count, procalcitonin, creatinine, and lactate did not differ significantly between MDR and susceptible infection (all *p* > 0.05), whereas glucose and bicarbonate were higher in the MDR group (Supplementary Table S2).

### 3.10. Outcome Analysis Among Infected Patients: Death Versus Survival

Among the 1413 infected ICU patients, 737 (52.2%) survived to hospital discharge and 676 (47.8%) died in hospital. Non-survivors were older than survivors (66.8 versus 60.9 years; *p* < 0.001) and had more frequent COVID-19 (19.7% versus 9.2%; *p* < 0.001). Sex distribution did not differ significantly between survivors and non-survivors (*p* = 0.387) (Table 6).

Admission laboratory variables showed a more severe physiological profile among non-survivors. Compared with survivors, non-survivors had higher white blood cell count (14.9 versus 13.0; *p* = 0.004), procalcitonin (9.3 versus 5.1; *p* < 0.001), PaCO2 (43.7 versus 41.2 mmHg; *p* < 0.001), creatinine (1.7 versus 1.3 mg/dL; *p* < 0.001), glucose (162.3 versus 151.8 mg/dL; *p* = 0.006), potassium (4.3 versus 4.1 mmol/L; *p* < 0.001), and lactate (3.2 versus 1.9 mmol/L; *p* < 0.001). Bicarbonate was lower in non-survivors than in survivors (25.0 versus 26.6 mmol/L; *p* < 0.001). CRP showed a numerical increase in non-survivors but did not reach statistical significance (141.1 versus 129.6; *p* = 0.068).

The distribution of infection sites differed by outcome mainly for bloodstream infection. Bloodstream infection was recorded in 212 non-survivors (31.4%) and 162 survivors (22.0%; *p* < 0.001). Ventilator-associated pneumonia was common in both groups and did not differ significantly (61.5% in non-survivors versus 58.8% in survivors; *p* = 0.311). Urinary tract infection also did not differ significantly between non-survivors and survivors (26.5% versus 23.5%; *p* = 0.214) (Table 6).

Several high-intensity interventions were more frequent among non-survivors. Mechanical ventilation was recorded in 99.9% of non-survivors and 97.2% of survivors (*p* < 0.001). Multiple vasoactive drugs were recorded in 75.0% of non-survivors and 55.9% of survivors (*p* < 0.001), cardiopulmonary resuscitation in 20.1% and 2.6% (*p* < 0.001), renal replacement therapy in 38.0% and 16.4% (*p* < 0.001), dialysis catheter insertion in 30.6% and 13.6% (*p* < 0.001), and treatment of acidosis or alkalosis in 40.2% and 14.2% (*p* < 0.001).

Some interventions were more frequent among survivors, including HFNC or CPAP support (23.3% versus 8.7%; *p* < 0.001), single vasoactive drug use (93.1% versus 79.1%; *p* < 0.001), enteral nutrition (92.4% versus 78.4%; *p* < 0.001), and procedures outside the ICU (61.5% versus 45.6%; *p* < 0.001). These differences describe the observed care patterns and do not establish whether the interventions themselves affected survival (Table 6).

Microbiological differences by survival status were limited. *Klebsiella pneumoniae* subsp. *pneumoniae* and *Pseudomonas aeruginosa* were recorded more frequently among survivors than non-survivors (14.7% versus 10.8%, *p* = 0.037; and 18.0% versus 11.5%, *p* = 0.001, respectively). Other selected pathogens and resistance phenotypes, including ESBL, MBL, NDM, VRE, MRSA, and MDR, did not differ significantly between survivors and non-survivors in the unadjusted infected-cohort analysis (Table 6).

### 3.11. Adjusted Mortality Models

Table 7 summarizes discrimination for the three complete-case logistic regression models. The whole-cohort model included 678 patients and had an accuracy of 0.724, specificity of 0.797, sensitivity of 0.640, and AUC of 0.790. VIFs ranged from 1.05 to 1.69, indicating no problematic multicollinearity. In this model (Table 8), MDR infection (adjusted OR 2.697, 95% CI 1.837–3.960; *p* < 0.001) and susceptible infection (adjusted OR 2.156, 95% CI 1.260–3.686; *p* = 0.005) were associated with higher odds of in-hospital death than no documented bacterial infection.

Other independently associated covariates in the whole-cohort model were age (OR 1.034 per year, 95% CI 1.021–1.048; *p* < 0.001), lactate at admission (OR 1.575, 95% CI 1.356–1.830; *p* < 0.001), creatinine at admission (OR 1.267, 95% CI 1.039–1.546; *p* = 0.020), and heart and/or respiratory failure (OR 0.626, 95% CI 0.432–0.909; *p* = 0.014). Sex, transfer from another hospital, pressure ulcers, renal failure, active malignancy, procalcitonin, and bicarbonate were not independently associated with mortality in this model.

The infected-cohort model included 352 patients and had an accuracy of 0.719, specificity of 0.685, sensitivity of 0.747, and AUC of 0.785; VIFs ranged from 1.09 to 1.81. MDR infection was not independently associated with death relative to susceptible infection (OR 1.086, 95% CI 0.611–1.932; *p* = 0.778). Mortality was associated with age (OR 1.037 per year, 95% CI 1.018–1.057; *p* < 0.001), lactate at admission (OR 1.327, 95% CI 1.063–1.657; *p* = 0.013), and lower pH at admission. After rescaling pH to a clinically interpretable 0.1-unit increment, the OR was 0.503 (95% CI 0.332–0.762; *p* = 0.001). Recent trauma and/or surgery showed an inverse association (OR 0.512, 95% CI 0.277–0.949; *p* = 0.033). The prognostic infected-cohort model included 348 patients, had an AUC of 0.820, and had VIFs ranging from 1.08 to 1.86. Overall, the bacterial infection category was associated with adjusted mortality in the complete-case whole-cohort model, whereas the MDR status did not distinguish mortality from susceptible infection within the infected cohort.

Among infected patients included in the complete-case analysis (*n* = 352), MDR bacterial infection was not independently associated with in-hospital mortality compared with antimicrobial-susceptible infection (adjusted OR 1.086, 95% CI 0.611–1.932; *p* = 0.778). Older age and higher lactate at admission were associated with higher odds of death, whereas recent trauma and/or surgery and a higher admission pH were associated with lower odds of death (Table 9).

In the expanded prognostic model of 348 infected patients, older age, higher lactate at admission, renal replacement therapy, and the use of multiple vasoactive drugs were associated with increased odds of in-hospital death. A higher admission pH was associated with lower odds of death. Ventilator-associated pneumonia, bloodstream infection, and urinary tract infection were not independently associated with mortality (Table 10).

## 4. Discussion

### 4.1. Summary

In this retrospective ICU cohort, bacterial infection was common and the operational MDR category accounted for most infections. Crude mortality was similar among patients with no documented infection, susceptible infection, and MDR infection. In complete-case adjusted analyses, both infection groups had higher odds of in-hospital death than the no-infection group; however, MDR status was not independently associated with mortality relative to susceptible infection among infected patients. MDR infection was instead characterized primarily by longer ICU exposure, greater organ-support requirements, and a distinct healthcare-associated microbiological profile.

### 4.2. Interpretation

The divergence between crude and adjusted estimates requires cautious interpretation. Crude mortality was high in all three groups, and the adjusted models were restricted to complete cases because several admission biomarkers were incompletely recorded. Consequently, the whole-cohort adjusted association may reflect both covariate adjustment and selection of patients with complete laboratory data. Within the infected cohort, the absence of an independent mortality difference between MDR and susceptible infection suggests that host severity, infection itself, infection site, timeliness of active therapy, and other unmeasured factors may contribute more strongly to mortality than the resistance category alone [4,5].

### 4.3. Integration

These findings are consistent with evidence that AMR imposes clinical and system-level costs that are not fully captured by mortality [1,14]. Resistant organisms may complicate empirical and definitive treatment, increase infection-control requirements, and coexist with prolonged exposure to invasive devices and organ support. The higher frequencies of *Klebsiella pneumoniae*, *Pseudomonas aeruginosa*, *Enterococcus* spp., and *Staphylococcus epidermidis* in the MDR group are compatible with a healthcare-associated microbiological profile [15,16,17,18]. However, the association between MDR infection and ICU length of stay is likely bidirectional: prolonged hospitalization increases the opportunity to acquire resistant organisms, whereas difficult-to-treat infection may itself delay recovery and discharge. The present observational design cannot determine the direction or magnitude of these pathways.

### 4.4. Implications

These results support reporting resistance status together with infection site, timing, organ-support exposure, and local microbiology rather than interpreting MDR status as an isolated mortality determinant. Infection-prevention programs should continue to prioritize artificial-airway, central-line, and urinary-catheter practices. Empirical antimicrobial decisions should incorporate local susceptibility data, previous antimicrobial exposure, transfer history, and the likely site and timing of infection, followed by prompt de-escalation when microbiological results become available. The observed association between bloodstream infection and mortality reinforces the importance of early recognition, source control, and prevention of catheter-related infection [3].

### 4.5. Limitations

Several limitations should be considered. First, the retrospective single-center design precludes causal inference and limits external generalizability, particularly because resistance epidemiology, laboratory coding, antimicrobial practice, and device utilization vary among ICUs. Residual confounding remains likely because severity scores such as APACHE II and SOFA, appropriateness and timing of antimicrobial therapy, source-control timing, and other potentially important variables were not consistently available. Indication bias may also affect associations involving antimicrobials, organ support, and invasive procedures.

Second, infection and MDR status were analyzed as fixed categories recorded during the ICU stay. Patients had to survive and remain hospitalized long enough for many healthcare-associated infections and resistant organisms to be detected, creating potential survivor and immortal-time bias [6,7,8]. The markedly longer ICU stay in the MDR group may therefore be a risk factor for, a consequence of, or both a risk factor for and a consequence of MDR infection. A Cox or multistate model with MDR status as a time-dependent exposure would better address this problem; however, phenotype-specific acquisition times were not retained in the analytical extract, so a valid time-updated analysis could not be performed.

Third, admission laboratory variables had substantial missingness, and complete-case modelling reduced the analytic samples to 678, 352, and 348 patients. Selection bias cannot be excluded, and no imputation was undertaken. The MDR classification was based on local laboratory resistance-phenotype coding because full agent-level susceptibility panels were unavailable for retrospective reconstruction; some local phenotype labels do not map one-to-one onto the Magiorakos antimicrobial categories. Polymicrobial infections and multiple isolates were classified according to the most resistant clinically relevant isolate, while pathogen and phenotype counts remained non-mutually exclusive. These features may introduce exposure misclassification and should be considered when interpreting the results.

## 5. Conclusions

In this single-center ICU cohort, the operational MDR category identified patients with longer ICU stays, prolonged organ-support exposure, and a distinctive healthcare-associated microbiological profile. In complete-case adjusted analyses, bacterial infection was associated with in-hospital mortality relative to no documented infection, whereas MDR status was not independently associated with death relative to susceptible infection among infected patients. The findings should be interpreted as descriptive and adjusted associations because the timing of MDR acquisition, residual confounding, and missing data preclude causal conclusions.

## Figures and Tables

**Table 1:** Baseline characteristics, admission variables, and outcomes according to bacterial infection category.

Characteristic	MDR Infection (*n* = 932)	Susceptible Infection (*n* = 481)	No Infection (*n* = 1913)	*p*-Value
Cohort distribution and outcomes				
Total N (%)	932 (28.0)	481 (14.5)	1913 (57.5)	
In-hospital death	451 (48.4)	225 (46.8)	871 (45.5)	0.354
Length of ICU stay, days, mean (SD)	25.2 (21.6)	15.5 (16.2)	10.0 (24.8)	<0.001
Baseline characteristics				
Age, years, mean (SD)	63.7 (16.2)	63.8 (16.3)	63.5 (16.8)	0.895
Female sex	337 (36.2)	189 (39.3)	755 (39.5)	0.219
Male sex	595 (63.8)	292 (60.7)	1158 (60.5)	0.219
Obesity by BMI	223 (23.9)	112 (23.3)	320 (16.7)	<0.001
COVID-19	170 (18.2)	31 (6.4)	154 (8.1)	<0.001
Antibiotic therapy within previous 3 months	30 (3.2)	14 (2.9)	30 (1.6)	0.011
Pressure ulcers	76 (8.2)	22 (4.6)	50 (2.6)	<0.001
Admission laboratory variables				
CRP at admission, mean (SD)	135.3 (97.2)	134.7 (102.0)	136.7 (99.9)	0.941
WBC at admission, mean (SD)	13.5 (8.8)	14.6 (15.2)	14.6 (12.5)	0.123
Procalcitonin at admission, mean (SD)	6.8 (14.5)	7.8 (17.2)	6.3 (13.0)	0.311
Interleukin-6 at admission, mean (SD)	373.2 (617.3)	348.8 (612.3)	527.2 (866.9)	0.089
Creatinine at admission, mg/dL, mean (SD)	1.5 (1.0)	1.5 (1.0)	1.7 (1.3)	0.014
Glucose at admission, mg/dL, mean (SD)	159.9 (48.4)	151.5 (48.2)	150.9 (49.2)	0.008
Bicarbonate at admission, mmol/L, mean (SD)	26.3 (4.9)	24.7 (5.0)	24.6 (6.0)	<0.001
Lactate at admission, mmol/L, mean (SD)	2.5 (2.5)	2.9 (3.9)	3.6 (4.5)	<0.001

Note. Values are mean (SD) or *n* (%), as appropriate. CRP, C-reactive protein; ICU, intensive care unit; MDR, multidrug-resistant; WBC, white blood cell count.

**Table 2:** Selected TISS-28 intervention duration and intervention occurrence according to bacterial infection category.

Characteristic	MDR Infection (*n* = 932)	Susceptible Infection (*n* = 481)	No Infection (*n* = 1913)	*p*-Value
TISS-28 intervention duration, days, mean (SD)				
Mechanical ventilation	22.7 (19.6)	13.3 (13.8)	8.5 (11.0)	<0.001
Artificial airway care	26.4 (23.1)	15.2 (15.6)	9.8 (13.2)	<0.001
Respiratory physiotherapy	19.1 (19.4)	12.1 (13.5)	4.9 (7.4)	<0.001
Single vasoactive drug	13.4 (13.6)	8.1 (9.0)	4.1 (6.4)	<0.001
Multiple vasoactive drugs	6.1 (8.8)	3.7 (6.1)	4.9 (9.4)	<0.001
Central venous catheter	27.3 (22.7)	16.3 (15.5)	10.4 (12.6)	<0.001
Renal replacement therapy	4.1 (10.0)	2.2 (6.4)	1.4 (4.4)	<0.001
Dialysis catheter insertion	0.3 (0.6)	0.2 (0.5)	0.1 (0.4)	<0.001
Parenteral nutrition	11.1 (17.5)	5.5 (10.8)	5.8 (12.2)	<0.001
Enteral nutrition	20.5 (20.4)	11.6 (13.6)	6.9 (11.5)	<0.001
Procedures outside the ICU	1.3 (2.3)	1.0 (1.6)	0.5 (1.0)	<0.001
TISS-28 intervention recorded at least once, *n* (%)				
Mechanical ventilation	920 (98.7)	471 (97.9)	1773 (92.7)	<0.001
Artificial airway care	922 (98.9)	472 (98.1)	1791 (93.6)	<0.001
Single vasoactive drug	830 (89.1)	391 (81.3)	1228 (64.2)	<0.001
Multiple vasoactive drugs	630 (67.6)	289 (60.1)	1193 (62.4)	0.006
Central venous catheter	924 (99.1)	477 (99.2)	1796 (93.9)	<0.001
Cardiopulmonary resuscitation	118 (12.7)	37 (7.7)	222 (11.6)	0.017
Renal replacement therapy	272 (29.2)	106 (22.0)	355 (18.6)	<0.001
Dialysis catheter insertion	232 (24.9)	75 (15.6)	244 (12.8)	<0.001
Parenteral nutrition	619 (66.4)	232 (48.2)	986 (51.5)	<0.001
Enteral nutrition	818 (87.8)	393 (81.7)	1228 (64.2)	<0.001
Procedures outside the ICU	515 (55.3)	246 (51.1)	583 (30.5)	<0.001

Note. TISS-28 duration variables represent the number of ICU days on which the intervention was recorded. Binary variables indicate whether the intervention occurred at least once during ICU stay.

**Table 3:** Concomitant ICU infection sites according to MDR versus susceptible bacterial infection.

Characteristic	MDR Infection (*n* = 932)	Susceptible Infection (*n* = 481)	Total Infected (*n* = 1413)	*p*-Value
Total N (%)	932 (66.0)	481 (34.0)	1413	
Ventilator-associated pneumonia, *n* (%)	545 (58.5)	304 (63.2)	849 (60.1)	0.097
Bloodstream infection, *n* (%)	320 (34.3)	54 (11.2)	374 (26.5)	<0.001
Urinary tract infection, *n* (%)	270 (29.0)	82 (17.0)	352 (24.9)	<0.001

Note. Values are *n* (%). Percentages were calculated within MDR and susceptible bacterial infection groups. BSI, bloodstream infection; ICU, intensive care unit; MDR, multidrug-resistant.

**Table 4:** Selected pathogen distribution according to MDR versus susceptible bacterial infection.

Selected Pathogen	MDR Infection (*n* = 932)	Susceptible Infection (*n* = 481)	Total Infected (*n* = 1413)	*p*-Value
Total *n* (%)	932 (66.0)	481 (34.0)	1413	
*Enterococcus faecalis*	216 (23.2)	45 (9.4)	261 (18.5)	<0.001
*Enterococcus faecium*	137 (14.7)	15 (3.1)	152 (10.8)	<0.001
*Escherichia coli*	117 (12.6)	88 (18.3)	205 (14.5)	0.005
*Hemophilus influenzae*	14 (1.5)	44 (9.1)	58 (4.1)	<0.001
*Klebsiella pneumoniae*	283 (30.4)	31 (6.4)	314 (22.2)	<0.001
*Klebsiella pneumoniae* subsp. *pneumoniae*	170 (18.2)	11 (2.3)	181 (12.8)	<0.001
*Pseudomonas aeruginosa*	159 (17.1)	52 (10.8)	211 (14.9)	0.002
*Staphylococcus aureus*	173 (18.6)	118 (24.5)	291 (20.6)	0.010
*Staphylococcus epidermidis*	145 (15.6)	13 (2.7)	158 (11.2)	<0.001
*Streptococcus pneumoniae*	6 (0.6)	15 (3.1)	21 (1.5)	0.001

Note. Values are *n* (%). Percentages were calculated within MDR and susceptible-infection groups. Pathogen categories were not mutually exclusive when more than one clinically relevant isolate was recorded. The complete distribution is provided in Supplementary Table S4.

**Table 5:** Recorded antimicrobial-resistance phenotype codes among patients in the operational MDR group.

Resistance Phenotype Code	Patients, *n* (%)
Total N (%)	932 (100.0)
ESBL	274 (29.40)
MBL	281 (30.15)
NDM	77 (8.26)
KPC	7 (0.75)
OXA-48	8 (0.86)
VIM	11 (1.18)
VRE	67 (7.19)
HLAR	197 (21.14)
HLARG	19 (2.04)
HLARS	7 (0.75)
MRSA	41 (4.40)
MLSBI	62 (6.65)
MLSBK	149 (15.99)
MDR	227 (24.36)

Note. Values are *n* (%). Percentages were calculated using the operational MDR group as the denominator. Resistance phenotype codes were not mutually exclusive. MSS was excluded because it is a methicillin-susceptibility code and was not used to define operational MDR status. ESBL, extended-spectrum beta-lactamase; HLAR, high-level aminoglycoside resistance; HLARG, high-level gentamicin resistance; HLARS, high-level streptomycin resistance; KPC, *Klebsiella pneumoniae* carbapenemase; MBL, metallo-beta-lactamase; MLSBI, inducible macrolide-lincosamide-streptogramin B resistance; MLSBK, constitutive macrolide-lincosamide-streptogramin B resistance; MRSA, methicillin-resistant *Staphylococcus aureus*; NDM, New Delhi metallo-beta-lactamase; OXA-48, oxacillinase-48-like carbapenemase; VIM, Verona integron-encoded metallo-beta-lactamase; VRE, vancomycin-resistant enterococci.

**Table 6:** Unadjusted prognostic comparison among infected ICU patients according to in-hospital mortality.

Characteristic	Survivors (*n* = 737)	Non-Survivors (*n* = 676)	Total Infected (*n* = 1413)	*p*-Value
Cohort distribution				
Total N (%)	737 (52.2)	676 (47.8)	1413	
Selected pathogens and resistance phenotypes				
*Klebsiella pneumoniae*	152 (20.6)	162 (24.0)	314 (22.2)	0.149
*Klebsiella pneumoniae* subsp. *pneumoniae*	108 (14.7)	73 (10.8)	181 (12.8)	0.037
*Pseudomonas aeruginosa*	133 (18.0)	78 (11.5)	211 (14.9)	0.001
*Staphylococcus aureus*	164 (22.3)	127 (18.8)	291 (20.6)	0.123
*Staphylococcus epidermidis*	91 (12.3)	67 (9.9)	158 (11.2)	0.172
ESBL	142 (19.3)	132 (19.5)	274 (19.4)	0.955
MBL	152 (20.6)	129 (19.1)	281 (19.9)	0.510
NDM	37 (5.0)	40 (5.9)	77 (5.4)	0.532
VRE	38 (5.2)	29 (4.3)	67 (4.7)	0.522
MRSA	19 (2.6)	22 (3.3)	41 (2.9)	0.550
MDR	110 (14.9)	117 (17.3)	227 (16.1)	0.252
Selected TISS-28 interventions recorded at least once, *n* (%)				
Mechanical ventilation	716 (97.2)	675 (99.9)	1391 (98.4)	<0.001
HFNC or CPAP support	172 (23.3)	59 (8.7)	231 (16.3)	<0.001
Single vasoactive drug	686 (93.1)	535 (79.1)	1221 (86.4)	<0.001
Multiple vasoactive drugs	412 (55.9)	507 (75.0)	919 (65.0)	<0.001
Cardiopulmonary resuscitation	19 (2.6)	136 (20.1)	155 (11.0)	<0.001
Renal replacement therapy	121 (16.4)	257 (38.0)	378 (26.8)	<0.001
Dialysis catheter insertion	100 (13.6)	207 (30.6)	307 (21.7)	<0.001
Treatment of acidosis/alkalosis	105 (14.2)	272 (40.2)	377 (26.7)	<0.001
Enteral nutrition	681 (92.4)	530 (78.4)	1211 (85.7)	<0.001
Procedures outside the ICU	453 (61.5)	308 (45.6)	761 (53.9)	<0.001
Clinical characteristics and admission variables				
Age, years, mean (SD)	60.9 (17.4)	66.8 (14.2)	63.8 (16.2)	<0.001
Female sex	266 (36.1)	260 (38.5)	526 (37.2)	0.387
Male sex	471 (63.9)	416 (61.5)	887 (62.8)	0.387
COVID-19	68 (9.2)	133 (19.7)	201 (14.2)	<0.001
Antifungal drug	215 (29.2)	176 (26.0)	391 (27.7)	0.209
Antibacterial drug	485 (65.8)	371 (54.9)	856 (60.6)	<0.001
Steroid therapy	260 (35.3)	197 (29.1)	457 (32.3)	0.016
Pressure ulcers	64 (8.7)	34 (5.0)	98 (6.9)	0.009
CRP at admission, mean (SD)	129.6 (91.5)	141.1 (105.7)	135.1 (98.8)	0.068
WBC at admission, mean (SD)	13.0 (7.7)	14.9 (14.3)	13.9 (11.4)	0.004
Procalcitonin at admission, mean (SD)	5.1 (12.6)	9.3 (17.6)	7.1 (15.4)	<0.001
PaCO_2_ at admission, mmHg, mean (SD)	41.2 (6.2)	43.7 (9.3)	42.5 (8.1)	<0.001
Creatinine at admission, mg/dL, mean (SD)	1.3 (0.9)	1.7 (1.1)	1.5 (1.0)	<0.001
Glucose at admission, mg/dL, mean (SD)	151.8 (40.7)	162.3 (54.2)	157.3 (48.4)	0.006
Potassium at admission, mmol/L, mean (SD)	4.1 (0.4)	4.3 (0.6)	4.2 (0.5)	<0.001
Bicarbonate at admission, mmol/L, mean (SD)	26.6 (4.1)	25.0 (5.6)	25.8 (5.0)	<0.001
Lactate at admission, mmol/L, mean (SD)	1.9 (1.4)	3.2 (3.8)	2.6 (3.0)	<0.001
Concurrent infections				
Ventilator-associated pneumonia	433 (58.8)	416 (61.5)	849 (60.1)	0.311
Bloodstream infection	162 (22.0)	212 (31.4)	374 (26.5)	<0.001
Urinary tract infection	173 (23.5)	179 (26.5)	352 (24.9)	0.214

Note. Values are mean (SD) or *n* (%), as appropriate. MDR, multidrug-resistant.

**Table 7:** Predictive performance of multivariable logistic regression models.

Model	Accuracy	Specificity	Sensitivity	AUC
Whole-cohort mortality model (*n* = 678)	0.724	0.797	0.640	0.790
Infected-cohort MDR versus susceptible model (*n* = 352)	0.719	0.685	0.747	0.785
Prognostic infected-cohort model (*n* = 348)	0.759	0.719	0.793	0.820

Note. Complete-case sample sizes are shown in the model labels. The probability cut-off was 0.5. AUC, area under the receiver-operating characteristic curve.

**Table 8:** Primary multivariable logistic regression model for in-hospital mortality in the total ICU cohort.

Predictor	Adjusted OR	95% CI	*p* Value
MDR bacterial infection versus no infection	2.697	1.837–3.960	<0.001
Susceptible bacterial infection versus no infection	2.155	1.260–3.686	0.005
Age, per year	1.034	1.021–1.048	<0.001
Sex	1.140	0.789–1.647	0.484
Transfer from another hospital within previous 6 months	0.844	0.393–1.810	0.663
Pressure ulcers	0.537	0.193–1.501	0.236
Renal failure	1.143	0.534–2.445	0.731
Heart and/or respiratory failure	0.626	0.432–0.909	0.014
Active malignancy	1.095	0.538–2.227	0.802
Lactate at admission	1.575	1.356–1.830	<0.001
Creatinine at admission	1.267	1.039–1.546	0.020
Procalcitonin at admission	1.008	0.994–1.022	0.252
Bicarbonate at admission	1.009	0.966–1.054	0.683

Note. The no-infection group was the reference category. The complete-case model included 678 patients. VIFs ranged from 1.05 to 1.69. Results are adjusted odds ratios with 95% confidence intervals; the outcome was in-hospital death.

**Table 9:** Secondary, multivariable logistic regression model for MDR versus susceptible infection among infected ICU patients.

Predictor	Adjusted OR	95% CI	*p* Value
MDR bacterial infection versus susceptible infection	1.086	0.611–1.932	0.778
Age, per year	1.037	1.018–1.057	<0.001
Sex	1.572	0.927–2.668	0.094
COVID-19	1.903	0.969–3.737	0.062
Antibiotic therapy within previous 3 months	2.130	0.455–9.965	0.337
Transfer from another hospital within previous 6 months	0.527	0.158–1.758	0.297
Recent trauma and/or surgical procedure	0.512	0.277–0.949	0.033
Pressure ulcers	0.672	0.195–2.320	0.530
Renal failure	2.078	0.693–6.236	0.192
Heart and/or respiratory failure	1.004	0.529–1.905	0.990
Lactate at admission	1.327	1.063–1.657	0.013
Procalcitonin at admission	1.016	0.996–1.036	0.119
Creatinine at admission	0.957	0.721–1.271	0.760
pH at admission, per 0.1-unit increase	0.503	0.332–0.762	0.001

Note. Susceptible bacterial infection was the reference category. The complete-case model included 352 infected patients. VIFs ranged from 1.09 to 1.81. Results are adjusted odds ratios with 95% confidence intervals; the outcome was in-hospital death. The pH estimate is reported per 0.1-unit increase.

**Table 10:** Prognostic multivariable logistic regression model for death versus survival among infected ICU patients.

Predictor	Adjusted OR	95% CI	*p* Value
Age, per year	1.035	1.015–1.056	<0.001
Sex	1.650	0.934–2.913	0.084
COVID-19	1.896	0.921–3.903	0.082
Antibiotic therapy within previous 3 months	1.223	0.231–6.483	0.813
Transfer from another hospital within previous 6 months	0.479	0.135–1.702	0.255
Recent trauma and/or surgical procedure	0.610	0.313–1.190	0.147
Pressure ulcers	0.489	0.121–1.970	0.314
Renal failure	2.148	0.689–6.696	0.187
Heart and/or respiratory failure	1.032	0.522–2.041	0.929
Lactate at admission	1.273	1.007–1.609	0.043
Procalcitonin at admission	1.009	0.988–1.030	0.396
Creatinine at admission	0.876	0.644–1.191	0.398
pH at admission, per 0.1-unit increase	0.542	0.350–0.838	0.006
Ventilator-associated pneumonia	1.122	0.586–2.151	0.728
Bloodstream infection	1.206	0.669–2.176	0.533
Urinary tract infection	0.992	0.565–1.744	0.978
White blood cell count at admission	1.034	0.997–1.072	0.073
Renal replacement therapy recorded at least once	2.056	1.106–3.823	0.023
Multiple vasoactive drugs recorded at least once	2.500	1.445–4.327	0.001

Note. This prognostic association model included 348 infected patients and was not designed as a causal model. VIFs ranged from 1.08 to 1.86. Results are adjusted odds ratios with 95% confidence intervals. The pH estimate is reported per 0.1-unit increase. Renal replacement therapy and multiple vasoactive drug use were analyzed as binary TISS-28 variables recorded at least once during the ICU stay.

## Data Availability

The data presented in this study are available upon reasonable request from the corresponding author. The data are not publicly available because of hospital data-protection policies.

## Supplementary Materials

The following supporting information can be downloaded at: https://www.mdpi.com/article/10.3390/jcm15156110/s1, Table S1: Secondary simplified comparison: bacterial infection versus no bacterial infection; Table S2: Subgroup comparison among infected patients: MDR versus sensitive bacterial infection; Table S3: VAP, UTI, and bloodstream infection according to infection and mortality status; Table S4: Complete pathogen distribution; Table S5: Missingness of principal variables; Table S6: Pairwise standardized mean differences; and the completed STROBE checklist.
