# Supplementary material for: Clinical Impact of Multidrug-Resistant Bacterial Infections in Critically Ill Patients: A Retrospective Cohort Study
Source: J Clin Med. 2026 Aug 6;15(15):6110. doi: 10.3390/jcm15156110 (PMC13467205; doi:10.3390/jcm15156110)
Supplement: Supplementary file 1 [file jcm-15-06110-s001.zip › JCM_MDR_clinical_impact_supplementary_revised.pdf]

Supplementary Materials

Clinical impact of multidrug-resistant bacterial infections in critically ill patients: a retrospective cohort study

Mateusz Bartoszewicz, Ewa Płonowska, Marta Krysik, Jerzy Robert Ładny and Sławomir Lech Czaban

Supplementary Table S1. Secondary simplified comparison: bacterial infection versus no bacterial infection.

Characteristic	Bacterial infection (n=1413)	No bacterial infection (n=1913)	Total cohort (N=3326)	p-value
Cohort distribution and outcomes				
Total N (%)	1413 (42.5)	1913 (57.5)	3326	
In-hospital death	676 (47.8)	871 (45.5)	1547 (46.5)	0.199
Length of ICU stay, days, mean (SD)	21.9 (20.5)	10.0 (24.8)	15.1 (23.8)	<0.001
Selected TISS-28 duration variables, days, mean (SD)				
Mechanical ventilation	19.5 (18.4)	8.5 (11.0)	13.2 (15.6)	<0.001
Artificial airway care	22.6 (21.5)	9.8 (13.2)	15.3 (18.4)	<0.001
Single vasoactive drug	11.6 (12.5)	4.1 (6.4)	7.3 (10.2)	<0.001
Multiple vasoactive drugs	5.3 (8.0)	4.9 (9.4)	5.1 (8.8)	0.195
Central venous catheter	23.5 (21.2)	10.4 (12.6)	16.0 (18.0)	<0.001
Renal replacement therapy	3.5 (9.0)	1.4 (4.4)	2.3 (6.8)	<0.001
Parenteral nutrition	9.2 (15.8)	5.8 (12.2)	7.3 (13.9)	<0.001
Enteral nutrition	17.5 (18.8)	6.9 (11.5)	11.4 (15.9)	<0.001
Selected TISS-28 interventions recorded at least once, n (%)				
Mechanical ventilation	1391 (98.4)	1773 (92.7)	3164 (95.1)	<0.001
Artificial airway care	1394 (98.7)	1791 (93.6)	3185 (95.8)	<0.001
Single vasoactive drug	1221 (86.4)	1228 (64.2)	2449 (73.6)	<0.001
Multiple vasoactive drugs	919 (65.0)	1193 (62.4)	2112 (63.5)	0.122
Central venous catheter	1401 (99.2)	1796 (93.9)	3197 (96.1)	<0.001
Cardiopulmonary resuscitation	155 (11.0)	222 (11.6)	377 (11.3)	0.606
Renal replacement therapy	378 (26.8)	355 (18.6)	733 (22.0)	<0.001
Parenteral nutrition	851 (60.2)	986 (51.5)	1837 (55.2)	<0.001
Enteral nutrition	1211 (85.7)	1228 (64.2)	2439 (73.3)	<0.001
Selected baseline and admission variables				
Age, years, mean (SD)	63.8 (16.2)	63.5 (16.8)	63.6 (16.6)	0.639
Female sex	526 (37.2)	755 (39.5)	1281 (38.5)	0.202
Male sex	887 (62.8)	1158 (60.5)	2045 (61.5)	0.202
Obesity by BMI	335 (23.7)	320 (16.7)	655 (19.7)	<0.001
COVID-19	201 (14.2)	154 (8.1)	355 (10.7)	<0.001
Antibiotic therapy within previous 3 months	44 (3.1)	30 (1.6)	74 (2.2)	0.004
Transfer from another hospital within previous 6 months	63 (4.5)	55 (2.9)	118 (3.5)	0.019

Characteristic	Bacterial infection (n=1413)	No bacterial infection (n=1913)	Total cohort (N=3326)	p-value
Pressure ulcers	98 (6.9)	50 (2.6)	148 (4.4)	<0.001
CRP at admission, mean (SD)	135.1 (98.8)	136.7 (99.9)	135.9 (99.3)	0.736
WBC at admission, mean (SD)	13.9 (11.4)	14.6 (12.5)	14.3 (12.0)	0.164
Procalcitonin at admission, mean (SD)	7.1 (15.4)	6.3 (13.0)	6.7 (14.3)	0.219
Interleukin-6 at admission, mean (SD)	366.2 (614.7)	527.2 (866.9)	436.3 (738.5)	0.029
Creatinine at admission, mg/dL, mean (SD)	1.5 (1.0)	1.7 (1.3)	1.6 (1.2)	0.004
Lactate at admission, mmol/L, mean (SD)	2.6 (3.0)	3.6 (4.5)	3.1 (3.9)	<0.001

Note. Values are mean (SD) or n (%), as appropriate. This table supports the secondary simplified analysis.

Supplementary Table S2. Subgroup comparison among infected patients: MDR versus susceptible bacterial infection.

Characteristic	MDR infection (n=932)	Susceptible infection (n=481)	Total infected (n=1413)	p-value
Cohort distribution and outcomes				
Total N (%)	932 (66.0)	481 (34.0)	1413	
In-hospital death	451 (48.4)	225 (46.8)	676 (47.8)	0.604
Time to death/observation, days, mean (SD)	23.3 (23.7)	13.5 (17.8)	20.0 (22.4)	<0.001
Selected TISS-28 duration variables, days, mean (SD)				
Mechanical ventilation	22.7 (19.6)	13.3 (13.8)	19.5 (18.4)	<0.001
Artificial airway care	26.4 (23.1)	15.2 (15.6)	22.6 (21.5)	<0.001
Single vasoactive drug	13.4 (13.6)	8.1 (9.0)	11.6 (12.5)	<0.001
Multiple vasoactive drugs	6.1 (8.8)	3.7 (6.1)	5.3 (8.0)	<0.001
Central venous catheter	27.3 (22.7)	16.3 (15.5)	23.5 (21.2)	<0.001
Renal replacement therapy	4.1 (10.0)	2.2 (6.4)	3.5 (9.0)	<0.001
Dialysis catheter insertion	0.3 (0.6)	0.2 (0.5)	0.3 (0.6)	<0.001
Parenteral nutrition	11.1 (17.5)	5.5 (10.8)	9.2 (15.8)	<0.001
Enteral nutrition	20.5 (20.4)	11.6 (13.6)	17.5 (18.8)	<0.001
Procedures outside the ICU	1.3 (2.3)	1.0 (1.6)	1.2 (2.1)	0.013
Selected TISS-28 interventions recorded at least once, n (%)				
Mechanical ventilation	920 (98.7)	471 (97.9)	1391 (98.4)	0.362
Artificial airway care	922 (98.9)	472 (98.1)	1394 (98.7)	0.322
Single vasoactive drug	830 (89.1)	391 (81.3)	1221 (86.4)	<0.001
Multiple vasoactive drugs	630 (67.6)	289 (60.1)	919 (65.0)	0.006
Central venous catheter	924 (99.1)	477 (99.2)	1401 (99.2)	1.000
Cardiopulmonary resuscitation	118 (12.7)	37 (7.7)	155 (11.0)	0.006
Renal replacement therapy	272 (29.2)	106 (22.0)	378 (26.8)	0.005
Dialysis catheter insertion	232 (24.9)	75 (15.6)	307 (21.7)	<0.001
Parenteral nutrition	619 (66.4)	232 (48.2)	851 (60.2)	<0.001
Enteral nutrition	818 (87.8)	393 (81.7)	1211 (85.7)	0.003
Procedures outside the ICU	515 (55.3)	246 (51.1)	761 (53.9)	0.157

Characteristic	MDR infection (n=932)	Susceptible infection (n=481)	Total infected (n=1413)	p-value
Selected clinical and laboratory variables				
Age, years, mean (SD)	63.7 (16.2)	63.8 (16.3)	63.8 (16.2)	0.953
Female sex	337 (36.2)	189 (39.3)	526 (37.2)	0.273
Male sex	595 (63.8)	292 (60.7)	887 (62.8)	0.273
Obesity by BMI	223 (23.9)	112 (23.3)	335 (23.7)	0.839
COVID-19	170 (18.2)	31 (6.4)	201 (14.2)	<0.001
Antifungal drug	287 (30.8)	104 (21.6)	391 (27.7)	<0.001
Antibacterial drug	546 (58.6)	310 (64.4)	856 (60.6)	0.037
Steroid therapy	321 (34.4)	136 (28.3)	457 (32.3)	0.022
Pressure ulcers	76 (8.2)	22 (4.6)	98 (6.9)	0.016
CRP at admission, mean (SD)	135.3 (97.2)	134.7 (102.0)	135.1 (98.8)	0.927
WBC at admission, mean (SD)	13.5 (8.8)	14.6 (15.2)	13.9 (11.4)	0.114
Procalcitonin at admission, mean (SD)	6.8 (14.5)	7.8 (17.2)	7.1 (15.4)	0.400
Creatinine at admission, mg/dL, mean (SD)	1.5 (1.0)	1.5 (1.0)	1.5 (1.0)	0.805
Glucose at admission, mg/dL, mean (SD)	159.9 (48.4)	151.5 (48.2)	157.3 (48.4)	0.041
Bicarbonate at admission, mmol/L, mean (SD)	26.3 (4.9)	24.7 (5.0)	25.8 (5.0)	<0.001
Lactate at admission, mmol/L, mean (SD)	2.5 (2.5)	2.9 (3.9)	2.6 (3.0)	0.101

Note. Values are mean (SD) or n (%), as appropriate. This table supports the infected-cohort subgroup analysis.

Supplementary Table S3. Distribution of VAP, UTI, and bloodstream infection according to bacterial infection status and in-hospital mortality.

Analysis	Characteristic	No/reference group	Yes/exposed group	Total	p-value
Bacterial infection status	Total N (%)	1913 (57.5)	1413 (42.5)	3326	
Bacterial infection status	Ventilator-associated pneumonia, n (%)	67 (3.5)	849 (60.1)	916 (27.5)	<0.001
Bacterial infection status	Urinary tract infection, n (%)	44 (2.3)	352 (24.9)	396 (11.9)	<0.001
Bacterial infection status	Bloodstream infection, n (%)	0 (0.0)	374 (26.5)	374 (11.2)	<0.001
In-hospital mortality	Total N (%)	1779 (53.5)	1547 (46.5)	3326	
In-hospital mortality	Ventilator-associated pneumonia, n (%)	462 (26.0)	454 (29.3)	916 (27.5)	0.033
In-hospital mortality	Urinary tract infection, n (%)	195 (11.0)	201 (13.0)	396 (11.9)	0.080
In-hospital mortality	Bloodstream infection, n (%)	162 (9.1)	212 (13.7)	374 (11.2)	<0.001

Note. In the bacterial infection status analysis, the no/reference group corresponds to no bacterial infection and the yes/exposed group corresponds to bacterial infection. In the in-hospital mortality analysis, the no/reference group corresponds to survivors and the yes/exposed group corresponds to non-survivors. BSI, bloodstream infection; UTI, urinary tract infection; VAP, ventilator-associated pneumonia.

Supplementary Table S4. Complete pathogen distribution according to MDR versus susceptible bacterial infection.

**Pathogen**	**MDR infection (n=932)**	**Susceptible infection (n=481)**	**Total infected (n=1413)**	**p-value**
Total N (%)	932 (66.0)	481 (34.0)	1413	
Enterobacter cloacae complex	17 (1.8)	20 (4.2)	37 (2.6)	0.015
Enterobacter cloacae	22 (2.4)	15 (3.1)	37 (2.6)	0.503
Enterococcus faecalis	216 (23.2)	45 (9.4)	261 (18.5)	<0.001
Enterococcus faecium	137 (14.7)	15 (3.1)	152 (10.8)	<0.001
Escherichia coli	117 (12.6)	88 (18.3)	205 (14.5)	0.005
Haemophilus haemolyticus	1 (0.1)	4 (0.8)	5 (0.4)	0.089
Haemophilus influenzae	14 (1.5)	44 (9.1)	58 (4.1)	<0.001
Haemophilus parahaemolyticus	2 (0.2)	6 (1.2)	8 (0.6)	0.038
Haemophilus parainfluenzae	4 (0.4)	2 (0.4)	6 (0.4)	1.000
Hafnia alvei	1 (0.1)	4 (0.8)	5 (0.4)	0.089
Klebsiella aerogenes (*Enterobacter aerogenes*)	6 (0.6)	3 (0.6)	9 (0.6)	1.000
Klebsiella oxytoca	10 (1.1)	7 (1.5)	17 (1.2)	0.713
Klebsiella pneumoniae	283 (30.4)	31 (6.4)	314 (22.2)	<0.001
Klebsiella pneumoniae subsp. pneumoniae	170 (18.2)	11 (2.3)	181 (12.8)	<0.001
Lactobacillus casei	0 (0.0)	1 (0.2)	1 (0.1)	0.736
Lactobacillus paracasei	2 (0.2)	0 (0.0)	2 (0.1)	0.787
Lactococcus garvieae	1 (0.1)	1 (0.2)	2 (0.1)	1.000
Lactococcus lactis	1 (0.1)	0 (0.0)	1 (0.1)	1.000
Coagulase-negative *Staphylococcus spp.*	55 (5.9)	25 (5.2)	80 (5.7)	0.674
Comamonas testosteroni	0 (0.0)	1 (0.2)	1 (0.1)	0.736
Corynebacterium jeikeium	0 (0.0)	1 (0.2)	1 (0.1)	0.736
Corynebacterium propinquum	0 (0.0)	1 (0.2)	1 (0.1)	0.736
Corynebacterium spp.	21 (2.3)	11 (2.3)	32 (2.3)	1.000
Corynebacterium striatum	4 (0.4)	2 (0.4)	6 (0.4)	1.000

Pathogen	MDR infection (n=932)	Susceptible infection (n=481)	Total infected (n=1413)	p-value
Cutibacterium acnes (*Propionibacterium acnes*)	1 (0.1)	2 (0.4)	3 (0.2)	0.559
Delftia acidovorans	0 (0.0)	1 (0.2)	1 (0.1)	0.736
Enterobacter aerogenes	4 (0.4)	1 (0.2)	5 (0.4)	0.848
Enterobacter cancerogenus	0 (0.0)	2 (0.4)	2 (0.1)	0.221
Enterobacter cloacae subsp. cloacae	1 (0.1)	1 (0.2)	2 (0.1)	1.000
Enterobacter hormaechei	11 (1.2)	3 (0.6)	14 (1.0)	0.473
Pseudomonas aeruginosa	159 (17.1)	52 (10.8)	211 (14.9)	0.002
Staphylococcus aureus	173 (18.6)	118 (24.5)	291 (20.6)	0.010
Staphylococcus auricularis	1 (0.1)	0 (0.0)	1 (0.1)	1.000
Staphylococcus capitis	3 (0.3)	2 (0.4)	5 (0.4)	1.000
Staphylococcus epidermidis	145 (15.6)	13 (2.7)	158 (11.2)	<0.001
Streptococcus pneumoniae	6 (0.6)	15 (3.1)	21 (1.5)	0.001
Streptococcus spp.	1 (0.1)	3 (0.6)	4 (0.3)	0.229

Note. Values are n (%). Percentages were calculated within MDR and susceptible-infection groups. Pathogen categories were not mutually exclusive when more than one clinically relevant isolate was recorded.

Supplementary Table S5. Missingness of principal variables in the whole cohort and infected cohort.

Variable	Available, whole cohort n (%)	Missing, whole cohort n (%)	Available, infected cohort n (%)	Missing, infected cohort n (%)
Age, years	3265 (98.2)	61 (1.8)	1413 (100.0)	0 (0.0)
Sex	3326 (100.0)	0 (0.0)	1413 (100.0)	0 (0.0)
Bacterial infection category	3326 (100.0)	0 (0.0)	1413 (100.0)	0 (0.0)
In-hospital mortality	3326 (100.0)	0 (0.0)	1413 (100.0)	0 (0.0)
Transfer from another hospital within previous 6 months	3326 (100.0)	0 (0.0)	1413 (100.0)	0 (0.0)
Pressure ulcers	3326 (100.0)	0 (0.0)	1413 (100.0)	0 (0.0)
Renal failure	3326 (100.0)	0 (0.0)	1413 (100.0)	0 (0.0)
Heart and/or respiratory failure	3326 (100.0)	0 (0.0)	1413 (100.0)	0 (0.0)
Active malignancy	3326 (100.0)	0 (0.0)	1413 (100.0)	0 (0.0)
COVID-19	3326 (100.0)	0 (0.0)	1413 (100.0)	0 (0.0)
Antibiotic therapy within previous 3 months	3326 (100.0)	0 (0.0)	1413 (100.0)	0 (0.0)
Recent trauma and/or surgical procedure	3326 (100.0)	0 (0.0)	1413 (100.0)	0 (0.0)
Lactate at admission	1285 (38.6)	2041 (61.4)	639 (45.2)	774 (54.8)
Creatinine at admission	1281 (38.5)	2045 (61.5)	637 (45.1)	776 (54.9)
Procalcitonin at admission	1700 (51.1)	1626 (48.9)	873 (61.8)	540 (38.2)
Bicarbonate at admission	1288 (38.7)	2038 (61.3)	640 (45.3)	773 (54.7)
pH at admission	1288 (38.7)	2038 (61.3)	640 (45.3)	773 (54.7)
WBC at admission	2456 (73.8)	870 (26.2)	1118 (79.1)	295 (20.9)
Ventilator-associated pneumonia	3326 (100.0)	0 (0.0)	1413 (100.0)	0 (0.0)
Bloodstream infection	3326 (100.0)	0 (0.0)	1413 (100.0)	0 (0.0)
Urinary tract infection	3326 (100.0)	0 (0.0)	1413 (100.0)	0 (0.0)
Renal replacement therapy	3326 (100.0)	0 (0.0)	1413 (100.0)	0 (0.0)
Multiple vasoactive drugs	3326 (100.0)	0 (0.0)	1413 (100.0)	0 (0.0)

Note. Percentages use N=3,326 for the whole cohort and n=1,413 for the infected cohort. Multivariable models used complete cases: n=678 for the whole-cohort mortality model, n=352 for the infected-cohort MDR-versus-susceptible model, and n=348 for the prognostic infected-cohort model. No imputation was performed.

Supplementary Table S6. Pairwise standardized mean differences for selected baseline and admission variables.

Variable	MDR vs susceptible	MDR vs no infection	Susceptible vs no infection
Age, years	-0.003	0.015	0.019
Female sex	-0.065	-0.068	-0.004
Obesity by BMI	0.015	0.180	0.164
COVID-19	0.364	0.305	-0.062
Antibiotic therapy within previous 3 months	0.018	0.108	0.091
Pressure ulcers	0.147	0.247	0.105
CRP at admission	0.006	-0.013	-0.019
WBC at admission	-0.092	-0.099	0.005
Procalcitonin at admission	-0.059	0.040	0.098
Interleukin-6 at admission	0.040	-0.205	-0.238
Creatinine at admission	-0.021	-0.169	-0.151
Glucose at admission	0.174	0.185	0.012
Bicarbonate at admission	0.319	0.305	0.016
Lactate at admission	-0.129	-0.297	-0.159

Note. SMDs are calculated as the first-listed group minus the second-listed group and are presented as signed values. Absolute SMDs of approximately 0.10, 0.20, and 0.50 are commonly interpreted as small, moderate, and large imbalances, respectively. SMDs are descriptive and do not replace adjusted analysis.
