# Supplementary material for: Clinical Impact of Multidrug-Resistant Bacterial Infections in Critically Ill Patients: A Retrospective Cohort Study
Source: J Clin Med. 2026 Aug 6;15(15):6110. doi: 10.3390/jcm15156110 (PMC13467205; doi:10.3390/jcm15156110)
Supplement: Supplementary file 1 [file jcm-15-06110-s001.zip › JCM_MDR_STROBE_checklist.pdf]

STROBE Statement - Checklist for Cohort Studies

Clinical impact of multidrug-resistant bacterial infections in critically ill patients: a retrospective cohort study

Section/topic	Item	STROBE recommendation	Reported in manuscript	Status
Title and abstract	1(a)	Indicate the study design with a commonly used term in the title or abstract.	Title; Abstract, Methods.	Reported
Title and abstract	1(b)	Provide an informative and balanced summary of what was done and found.	Structured Abstract.	Reported
Background/rationale	2	Explain the scientific background and rationale for the investigation.	Introduction, paragraphs 1-3.	Reported
Objectives	3	State specific objectives, including prespecified hypotheses.	Introduction, final paragraph.	Reported
Study design	4	Present key elements of study design early in the paper.	Methods, Section 2.1.	Reported
Setting	5	Describe setting, locations, and relevant dates, including recruitment, exposure, follow-up, and data collection.	Methods, Sections 2.1-2.3.	Reported
Participants	6(a)	Give eligibility criteria and sources/methods of participant selection; describe follow-up.	Methods, Section 2.2; Figure 1.	Reported
Participants	6(b)	For matched studies, give matching criteria and numbers exposed/unexposed.	Not applicable; no matching was performed.	Reported
Variables	7	Clearly define outcomes, exposures, predictors, confounders, and effect modifiers; provide diagnostic criteria.	Methods, Sections 2.3-2.7.	Reported
Data sources/measurement	8	For each variable, give sources of data and details of methods of assessment; describe comparability between groups.	Methods, Sections 2.1, 2.3, 2.5-2.7.	Reported
Bias	9	Describe efforts to address potential sources of bias.	Methods, Sections 2.2, 2.8, 2.9; Discussion, Limitations.	Reported
Study size	10	Explain how the study size was arrived at.	Methods, Section 2.2; all eligible index admissions during the study interval.	Reported
Quantitative variables	11	Explain how quantitative variables were handled;	Methods, Sections 2.5, 2.7-2.9.	Reported

Section/topic	Item	STROBE recommendation	Reported in manuscript	Status
		describe groupings.		
Statistical methods	12(a)	Describe all statistical methods, including methods used to control for confounding.	Methods, Section 2.9.	Reported
Statistical methods	12(b)	Describe methods used to examine subgroups and interactions.	Methods, Section 2.9; Results, Sections 3.8-3.10.	Reported
Statistical methods	12(c)	Explain how missing data were addressed.	Methods, Section 2.8; Supplementary Table S5.	Reported
Statistical methods	12(d)	If applicable, explain how loss to follow-up was addressed.	Not applicable; in-hospital outcome data were complete.	Reported
Statistical methods	12(e)	Describe sensitivity analyses.	No formal sensitivity analysis; limitations of fixed exposure classification are discussed.	Reported
Participants	13(a)	Report numbers at each stage of the study.	Figure 1; Results, Section 3.1.	Reported
Participants	13(b)	Give reasons for non-participation at each stage.	Methods, Section 2.2; repeated ICU hospitalizations excluded by design.	Reported
Participants	13(c)	Consider use of a flow diagram.	Figure 1.	Reported
Descriptive data	14(a)	Give characteristics of study participants and information on exposures and potential confounders.	Table 1; Supplementary Tables S1, S2, and S6.	Reported
Descriptive data	14(b)	Indicate number of participants with missing data for each variable.	Supplementary Table S5.	Reported
Descriptive data	14(c)	Summarize follow-up time.	ICU length of stay and time to death/observation: Table 1 and Supplementary Table S2.	Reported
Outcome data	15	Report numbers of outcome events or summary measures over time.	Results, Sections 3.3 and 3.10; Tables 1 and 6.	Reported
Main results	16(a)	Give unadjusted and adjusted estimates with precision; identify confounders included.	Results, Sections 3.3 and 3.11; Tables 8-10.	Reported
Main results	16(b)	Report category boundaries when continuous variables were categorized.	Not applicable to principal continuous predictors; pH rescaled per 0.1 unit.	Reported

Section/topic	Item	STROBE recommendation	Reported in manuscript	Status
Main results	16(c)	Consider translating relative risk into absolute risk for a meaningful period.	Absolute mortality risk differences with 95% CIs: Results, Sections 3.3, 3.8, and 3.9.	Reported
Other analyses	17	Report other analyses, including subgroup and sensitivity analyses.	Results, Sections 3.8-3.10; Supplementary Tables S1-S3 and S6.	Reported
Key results	18	Summarize key results with reference to study objectives.	Discussion, Section 4.1.	Reported
Limitations	19	Discuss limitations, including potential bias or imprecision and direction/magnitude.	Discussion, Section 4.5.	Reported
Interpretation	20	Provide cautious overall interpretation considering objectives, limitations, multiplicity, and other evidence.	Discussion, Sections 4.2-4.5; Conclusions.	Reported
Generalisability	21	Discuss generalisability of results.	Discussion, Section 4.5.	Reported
Funding	22	Give source of funding and role of funders.	Funding statement.	Reported

Note. The checklist uses section and table locations because pagination may change during editorial typesetting.
